# Ecology of *Rhodnius robustus* Larrousse, 1927 (Hemiptera, Reduviidae, Triatominae) in *Attalea* palm trees of the Tapajós River Region (Pará State, Brazilian Amazon)

**DOI:** 10.1186/1756-3305-7-154

**Published:** 2014-04-01

**Authors:** Fernando Braga Stehling Dias, Marion Quartier, Liléia Diotaiuti, Guy Mejía, Myriam Harry, Anna Carolina Lustosa Lima, Robert Davidson, Frédéric Mertens, Marc Lucotte, Christine A Romaña

**Affiliations:** 1 Laboratório de Triatomíneos e Epidemiologia da Doença de Chagas, Centro de Pesquisa René Rachou, Av Augusto de Lima, 1715 Barro Preto, Belo Horizonte, MG CEP 30190-002, Brazil; 2 LEGS, Laboratoire Evolution Génome et Spéciation UPR 9034, DEEIT - Diversité, Ecologie et Evolution des Insectes Tropicaux, CNRS, Avenue de la Terrasse, Bâtiment 13, Boîte Postale, 191198 Gif-sur-Yvette, France; 3 Laboratoire Ecologie et Evolution des Parasites, Institut de Biologie, Université de Neuchâtel, Rue Emile Argand 11 2000, Neuchâtel, Switzerland; 4 Centro de Investigaciones Médicas - CIBM, Universidad Simón Bolívar, Carrera 59 No. 59-92, A.A. 50595, Barranquilla, Colombia; 5 LEGS, UPR9034 CNRS-IRD-Paris Sud, Av de la Terasse, BP1, 91198 Gif-sur-Yvette/Université Paris Sud, UFR de Sciences, 91400 Orsay, France; 6 Centro de Pesquisa em Biotecnologia, Rua Juramento, 1464, Unidade Antônio Mourão, 3º andar Saudade, Belo Horizonte, MG CEP 30.285-000, Brazil; 7 GÉOTOP & Institut des Sciences de l’Environnement, Université du Québec à Montréal, C.P. 8888, succ. Centre-Ville, H3C 3P8 Montréal, Québec, Canada; 8 Biodôme de Montréal, Canada, 4777, Avenue Pierre-De Coubertin, Montréal H1V 1B3, Québec, Canada; 9 Centro de Desenvolvimento Sustentável, Universidade de Brasília, Campus Universitário Darcy Ribeiro - L3 Norte / Gleba A, Bloco C, 70910-900 Brasília, DF, Brazil; 10 Université Paris Descartes/PRES Sorbonne Paris Cité. 19 rue de Dantzig, Paris 75015, France

**Keywords:** *Rhodnius robustus*, Palm trees, Tapajós Region, *Trypanosoma cruzi*, *Trypanosoma rangeli*, Amazon Region, Brazil

## Abstract

**Background:**

The rising number of acute cases of Chagas disease in the State of Pará, reported in the past two decades, has been associated, in part, with the ingestion of juice of local palm tree fruits, mainly açaí berry and bacaba. Near the study area, in Santarém, Pará State, an outbreak of Chagas disease has been notified and investigations suggest the consumption of bacaba juice as the main source of infection with *T. cruzi*. The purpose of this study is to assess the aspects associated to the ecology of *Rhodnius robustus* in palm trees of three communities of the Tapajós region, in the State of Pará, Brazil.

**Methods:**

Palm trees were cut down and dissected to search for triatomines. DNA from triatomines was extracted to investigate natural infection by *Trypanosoma cruzi* and *T. rangeli*. For statistical analyzes, data from infestation of palm trees, as well as the rates of natural infection by *T. cruzi* and *T. rangeli* were compared by Chi-square test. Triatomine density values were analyzed by the nonparametric Kruskal Wallis test and then comparisons between each pair of variables were made by the Mann–Whitney test assuming a confidence interval of 95%.

**Results:**

We dissected 136 palm trees, 60 at the end of the rainy period and 76 at the end of the dry period. Seventy-three of them (53.7%) were infested with triatomines and three species were found, namely: *Rhodnius robustus*, *Rhodnius pictipes* and *Panstrongylus lignarius*. We collected 743 triatomines, and *R. robustus* was predominant (n = 739). The identification of natural infection of the insects by trypanosomatids revealed that 125 triatomines were infected by *T. cruzi*, 69 by *T. rangeli* and 14 presented both parasites, indicating the presence of mixed infection in the same vector.

**Conclusion:**

The results suggest that São Tomé is the community with greater density of triatomines and infestation of palm trees; also, it demonstrates the existence of an intense sylvatic cycle in the region, which demands intensive surveillance to prevent human transmission.

## Background

Approximately 100 years after its discovery, Chagas disease remains a serious public health problem in Latin America. It is estimated that this parasitic infection affects about 7.7 million people and approximately 109 million of them live in risk areas [1], and may be infected with the etiologic agent, the flagellate protozoan *Trypanosoma (Schyzotrypanum) cruzi* Chagas, 1909.

The main mechanism of transmission of the *T. cruzi* parasite to human populations is still through the feces of infected triatomines. Thereby, the scenario that stands out is the one of characteristic domiciliary transmission demonstrated by *R. prolixus* in Colombia, Venezuela and some Central American countries, with the establishment of large colonies in intradomiciliary habitats [2]. Outside the domiciliary scenario, many exclusively wild species of triatomines maintain the enzootic cycle of the *T. cruzi* in nature. A classic example is the species of the genus *Rhodnius*, which live in various species of palm trees [3-7].

In recent years, the Brazilian Amazonian Region has been drawing attention due to the constant occurrence of acute outbreaks of Chagas disease. The first autochthonous human case in the Amazon Region was registered in 1969, in Belém, Pará [8]. Recently, about 600 new acute cases of Chagas disease have been registered in the Brazilian Amazon [9]. In the State of Pará, Brazil, in a very short period, from January to November 2006, approximately 178 acute cases have been reported, which makes the region an emerging area for American Trypanosomiasis [10].

The process of occupation of the Brazilian Amazon began in the seventies with logging and the arrival of thousands of people from the Brazilian northeast [11]. Therefore, the different uses of the Amazonian territory resulted in a mosaic of heterogeneous landscapes, with the presence of slash and burn agriculture for subsistence farming and, recently, with large areas of cattle ranching and soy plantation. In some regions, a consequence of this process is the proliferation and the constant presence of palm trees. With regard to the mechanisms of extradomiciliary vector transmission, the *Attalea* genus palm trees are considered natural ecotopes for species of *Rhodnius* and it is possible to consider them as an ecological indicator of epidemiological risk for Chagas disease [5]. These palm trees, in addition to being natural triatomine ecotopes, have fruits and leaves that are used by many populations, as already demonstrated in other regions of Brazil [6]. Also, the *Attalea* genus palm tree is an important extractive source in Brazil [12].

The purpose of this study was to investigate the infestation of palm trees by the triatomines in three communities of the middle Tapajos Region, Pará, Brazil and to study the aspects related to ecology of *R. robustus*, as well as to determine the index of infection by trypanosomatids.

## Methods

### Area of study

In this study, three communities located near the middle Tapajós River, Pará, Brazil were investigated. The region of the study is part of the Amazonian biome and has a humid equatorial climate. The average temperature exceeds 25°C and it has two well-defined seasons with a dry period and a rainy period. From December to March, the precipitation index can exceed 400 mm, while in September, it does not reach 40 mm. The following communities were chosen for being heterogeneous in comparison to the landscape and for being different with regard to time of occupation of the area, origin of the population, anthropization levels and the occurrence of different species of palm trees.

### Araipá

Community near Araipá Lake comprised of 47 families, with a total of 198 inhabitants (93 women and 105 men). In the surroundings of the Araipá community, there is a predominance of inajá palm trees [*Attalea maripa* (Aubl.) Mart.], although the tucumã palm tree (*Astrocaryum aculeatum* G. Mey.) is also present. Most of the community land was deforested to serve as pasture for cattle and plantations, mainly of cassava and rice.

### São Tomé

Community nears the lake; it is located very close to the main bed of the Tapajós River. São Tomé is formed by a community of native families of the Tapajós River Region, comprising 25 houses and 106 inhabitants (45 women and 61 men). There is the constant presence of three species of the *Attalea* genus palm trees in this community: inajá, babaçu *(A. speciosa Mart.)* and uricuri (*A. phalerata* Mart. ex Spreng.). Within the forest fragments, there are bacaba (*Oenocarpus bacaba* Mart.) palm trees, and also macaúba [*Acrocomia aculeata* (Jacq.) Lodd. ex Mart.] palm trees, but in small quantities. Electricity is provided through diesel oil generators and most houses are built of wood with beaten clay floors and asbestos roofs. Only one house is made of masonry construction. Cultivation of the plantations is intended for subsistence and, on some occasions, for sale in nearby towns.

### Nova Estrela

**Figure 1:**
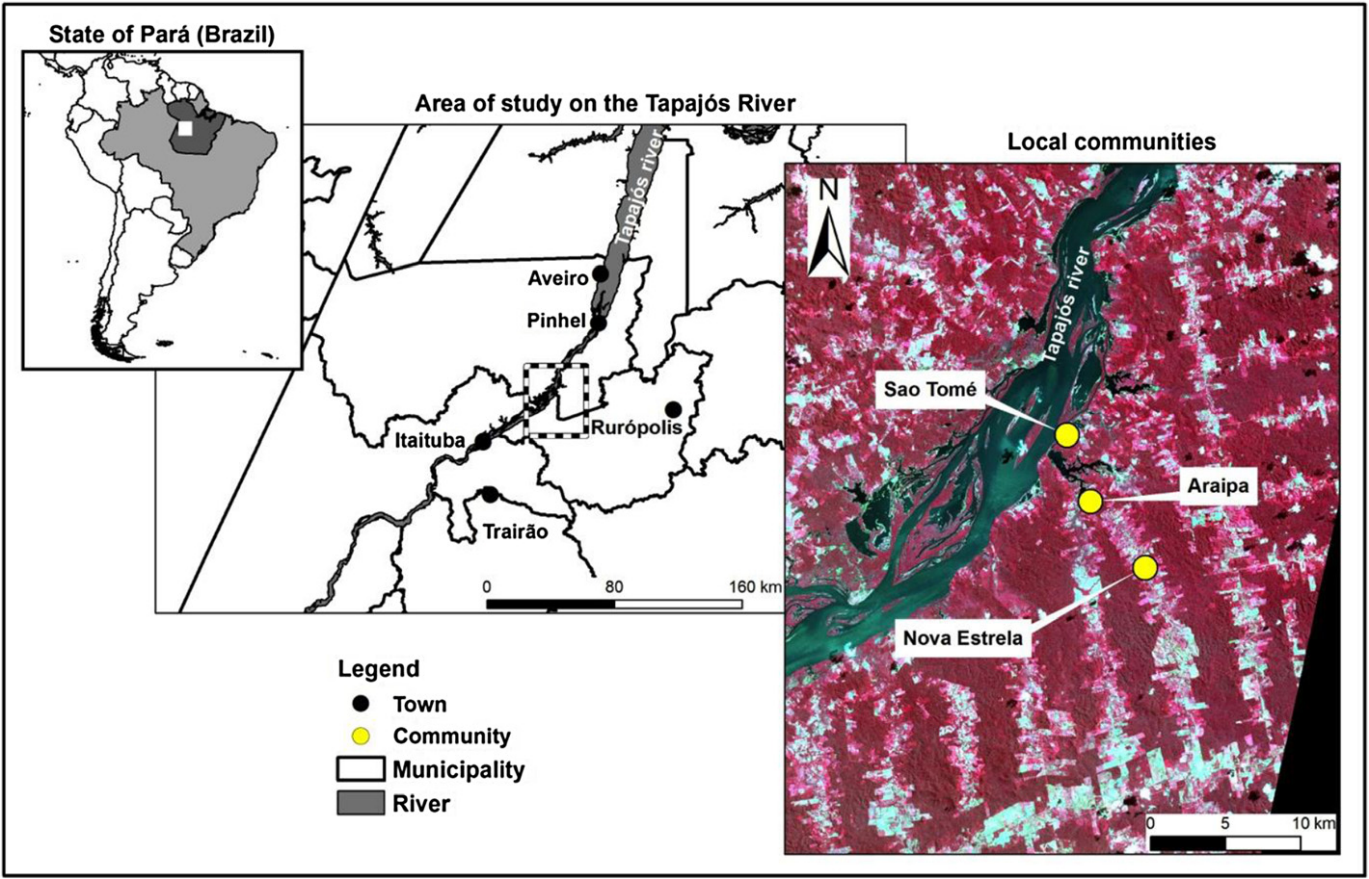
Localization of the three communities surveyed in the Tapajós Region, Pará State, Brazilian Amazon (Courtesy, by Delaitre E., UMR ESPACE-DEV, IRD).

Comprised of 42 families and 160 inhabitants (74 women and 86 men). This community is not on a riverside and is located at Km 60 of the Trans-Amazonian Highway, a difficult place to access during the rainy period. In this community, the majority of residents are from the State of Maranhão, some people who also live in the community are from Pará or natives of the region. In Nova Estrela, there is predominance of the *Attalea* genus palm tree, although there is a secondary presence of buriti (*Mauritia flexuosa* L.). Nova Estrela is part of the municipality of Rurópolis and has electric power lines provided by the Brazilian Government. As well as in Araipá and São Tomé, the houses are usually made of wood, although wattle and daub houses can be found, but in lesser numbers. Figure 1 shows the location of the communities surveyed.

### Collection of the triatomines

We conducted two collections of triatomines. The first, in the period from September 15 to October 8, 2008, period corresponding to the end of the dry season in the region; the second, from May 08 to 27, 2009, which coincides with the end of the rainy season. In the Nova Estrela community, the collections were made only at the end of the dry period, due to access difficulties. The other two communities were investigated in two field site trips.

The triatomines were collected through the dissection of palm trees [4,6] after authorization of the Brazilian Institute of Environment and Renewable Natural Resources (IBAMA, license number 16485–2). The palm trees were identified through systematic keys [13-15] and also through the use of the popular knowledge of local residents. An infested palm tree was defined as a palm tree containing one specimen of triatomine, eggs or even triatomine exuvia.

### Morphological and molecular identification of the insects collected

The triatomines collected were identified by their morphology according to the classification keys proposed [3]. In addition, a sample of insects was molecularly identified [16]. Two primers were used to amplify part of the conserved region of the cytochrome B gene (mtDNA) of *Triatoma dimidiata*. CYTB7432F, 5′- GGACG(AT)GG(AT)ATTTATTATGGATC-3′, and CYTB7433R, 5′- GC(AT)CCAATTCA(AG)GTTA(AG)TAA-3′ [17], being the forward primer labeled with fluorescein. The reagents used in the PCR were: 5 μL of 5X Green GoTaq® Flexi Buffer (Promega), 3 mM MgCl2, 200 μM dNTP, 10 μM of each primer, 1 U of GoTaq DNA polymerase (Promega) and 1 μl of “A” template DNA (see Section Controls used in PCR reactions below), resulting in total volume of 25 μl/reaction. The amplified product was subjected to 1% agarose gel electrophoresis, stained with ethidium bromide and the amplified fragments viewed with an UV lamp. The amplified product was purified using the Kit Wizard SV Gel and PCR Clean-up System (Promega) and dosed in a Nanodrop 2000c Spectrophotometer (Thermo Fisher Scientific Inc.). The forward primer was sequenced in the automatic sequencer ABI PRISM 3130 Genetic Analyzer, (Applied Biosystem, UK). The sequences obtained were compared with sequences deposited in genbank using the BLAST tool.

### *Trypanosoma* infection and hemolymph examination

All insects that arrived alive at the Laboratório de Triatomíneos e Epidemiologia da Doença de Chagas/FIOCRUZ (LATEC) were submitted to parasitological examination and hemolymph examination to confirm the presence of trypanosomatids.

Fresh feces of collected triatomines were diluted in a drop of saline solution at 0.9% and placed on a slide under a cover slip. For the hemolymph examination, one leg of the triatomines was cut with dissecting scissors and a micro drop of the hemolymph (approximately 10 μl) was placed on a slide under a cover slip. In both examinations, the material was observed in an optical microscope at a 160x magnification. The triatomines that presented flagellate forms of trypanosomatids were used for the isolation of the strain, according to the methodology previously described [18].

### Extraction of the triatomines DNA

The DNA of the triatomines was extracted using the kit DNease® Blood Tissue & Kit (Qiagen, USA), following the manufacturer’s recommendations. The extraction scheme used is as follows: for first and second stage nymphs, the DNA was extracted from the whole insect, named “DNA AB”; for third stage nymphs to adult, two legs and the chest of the triatomines were macerated together, and the extracted DNA was used for the study of infection by *T. rangeli*, named “DNA A”; for the study of infection by *T. cruzi*, the wings and upper abdominal cuticle were removed, and the final portion of the abdomen was cut in order to extract the DNA. Triatomines that were engorged, with a large amount of blood, had a small amount of the intestinal content removed with tweezers and the material was used for the extraction of DNA. This DNA was intended to the study of infection by *T. cruzi* and called “DNA B”.

### Molecular characterization of trypanosomatids

#### Identification of the natural infection by *Trypanosoma rangeli* in the insects collected

The identification of the natural infection by *T. rangeli* in the insects collected was based on molecular typing of a repetitive gene of small nucleolar RNA Cl1 (sno-RNA-Cl1) that amplifies a fragment of 620bp [19,20]. Reagents used in the PCR were: 1 μL of 5X Green GoTaq® Flexi Buffer (Promega), 1 mM MgCl2, 200 μM dNTP, 2.5 μM of each primer, 0.5 U of GoTaq DNA polymerase (Promega) and 2 μl of DNA “A”, resulting in total volume of 10 μl/reaction. PCR conditions are listed in Table 1.

**Table 1:** The thermal profile for each polymerase chain reaction, with the used primers

**Primers**	**Target**	**Thermal profile**
TrF and TrR2^a^	sno-RNA-Cl1 *T. rangeli*	95°C/5 min; 15 cycles of 95°C/30 s, 63°C/1 min, 72°C/30 s, 20 cycles of 95°C/30 s, 60°C/1 min, 72°C/30 s and 72°C/5 min.
TC, TC1 and TC2^b^	Mini-exon gene *T. cruzi*	94°C/5 min, 61°C/30 s, 72°C/30 s, 15 cycles of 94°C/1 min, 61°C/30 s, 72°C/30 s and 94°C/1 min, 61°C/30 s, 72°C/10 min.

^a^[20].

^b^Thermal profile modified [21].

#### Identification of the natural infection by *Trypanosoma cruzi* in the insects collected

The molecular typing of *T. cruzi* was based on the amplification of part of the intergenic spacer of the mini-exon gene. The expected fragment size varies between 300 and 350 bp [21]. The reagents used in the PCR were: 2.5 μL of 5X Green GoTaq® Flexi Buffer (Promega), 0.75 mM MgCl2, 200 μM dNTP, 2.5 μM of each primer, 1 U of GoTaq DNA polymerase (Promega) and 2–3 μl of DNA “B”, resulting in total volume of 25 μl/reaction. The PCR condition is described in Table 1. The amplified product was subjected to 1.5% agarose gel electrophoresis stained with ethidium bromide and the fragments were viewed using an UV photodocumentation system.

### Controls used in PCR reactions

Positive and negative controls were used in all *T. rangeli* and *T. cruzi* PCR reactions. For the positive control of *T. cruzi*, second stage nymphs of *Rhodnius prolixus* Stål, 1859 were orally infected in the lab using an artificial feeder with the CL strain (approximately 1×10^7^ parasites/insect). The insects were kept in the LATEC. When the insects reached the third stage, they were examined to confirm the infection and, upon reaching the fourth stage, the nymphs were left fasting until they died in order to simulate the same mortality of insects collected in the field. The DNA of these insects also served as negative control for the reactions of *T. rangeli*.

For *T. rangeli* positive control, fourth stage nymphs of *R. prolixus* were infected in the laboratory through an intrafemural inoculum of 50 parasites/insect of the Choachi strain. Seven days after the inoculum, the insects’ hemolymph was examined in order to verify the infection. As with insects infected with *T. cruzi*, the insects infected with *T. rangeli* were left fasting until their death. After they died, the insects were transferred to 1.5 mL tubes containing alcohol. The triatomines used as controls for the PCR reactions came from the LATEC. The DNA of these insects also served as negative control for the reactions of *T. cruzi*. In addition to these controls, one type of negative control, consisting in the PCR reaction mix without addition of DNA template, was used in all the reactions.

### Statistical analysis

The data obtained from the infestation of palm trees, as well as the rates of natural infection with *T. cruzi* and *T. rangeli* were compared by the Chi-square test. On the other hand, Triatomine density values were analyzed by the nonparametric Kruskal-Wallis test and then comparisons between each pair of variables were made using the Mann–Whitney test assuming a confidence interval of 95%.

## Results

### Infestation of palm trees

We dissected 136 palm trees of different genera and species. In the first collection, at the end of the dry period, 76 palm trees were dissected and, at the end of the rainy period, 60 palm trees. All the palm trees dissected were from four species, namely *A. speciosa*, *A. maripa*, *A. phalerata* and *A. aculeatum*.

At the end of the dry period, 15 of the 21 *A. speciosa* palm trees surveyed were infested by triatomines of the *Rhodnius* genus; 15 of the 36 *A. maripa* palm trees presented triatomines; 11 of the 16 *A. phalerata* palm trees were infested by *Rhodnius* and none of the three tucumã palm trees dissected presented kissing bugs. Statistical results showed significant infestation differences between the palm trees species (p = 0.045), and the global index of infestation in 2008 was 53.9%.

In 2009, we dissected 60 palm trees: 12 babaçus (83.3% infested), 38 inajás (34.2% infested) and 10 uricuris (90% infested). As in 2008, the statistical results for 2009 showed significant differences between palm tree species (p < 0.05). In this period, the global index of infestation was 53.3% and, therefore, summing the collections from both years, 53.7% of the palm trees surveyed were infested by triatomines.

In the global comparison of the communities, the lowest infestation index was at Araipá, with 26.3%, and the greatest infestation was found in the community of São Tomé with 68.5%. Table 2 presents data of infestation of palm trees by triatomines, community and collection period.

**Table 2:** Infestation by triatomines of palm trees surveyed according to the community, collection year

**Palm tree**	**2008**						**2009**				**Total**	
	**Araipá**		**Nova estrela**		**São tomé**		**Araipá**		**São tomé**			
	**S**	**I**	**S**	**I**	**S**	**I**	**S**	**I**	**S**	**I**	**S**	**I**
*Attalea speciosa*	-	-	7	3 (42.9)	14	12 (85.7)	-	-	12	10 (83.3)	33	25 (75.8)
*Attalea maripa*	17	6 (35.3)	4	1 (25)	15	8 (53.3)	18	4 (22.2)	20	9 (45)	74	28 (38.8)
*Astrocaryum aculeatum*	3	0	-	-	-	-	-	-	-	-	3	0
*Attalea phalerata*	-	-	14	9 (64.3)	2	2 (100)	-	-	10	9 (90)	26	20 (77)
Total	20	6 (30)	25	13 (52)	31	22 (71)	18	4 (22.2)	42	28 (66.7)	136	73 (53.7)

S = Number of palm trees surveyed.

I (%) = number of palm trees infested.

- = palm tree not surveyed or nonexistent in the collection site.

Although we did not perform intradomiciliary collection, disposable plastic pots were distributed to the population. Any insect found inside the houses was collected, stored and sent to the LATEC. Several species of insects were found, including some specimens of the Reduviidae family, but they were all predators. However, one of the collected insects drew our attention, namely a *R. robustus* female without stomach contents, indicating prolonged fasting in the living room wall of a house in the community of Nova Estrela during the second half of 2010.

### Triatomine density

In total, 743 triatomines were collected, including 120 adults and 623 nymphs (Table 3). Out of the 120 adults, 85 were males (70.8%) and were 35 females (29.2%). During the first fieldtrip, in 2008, we collected 424 triatomines, 359 (84.7%) nymphs and 65 (15.3%) adults belonging to three species: *R. robustus* (n = 739), *R. pictipes* (n = 3) *and Panstrongylus lignarius* (n = 1). Statistical results did not demonstrate significant differences in the triatomine density between the three communities surveyed (Kruskal-Wallis, p = 0.105), however, the results obtained through the comparison between the communities showed a difference only between São Tome and Araipá, with greater triatomine density found in São Tomé palm trees (Mann-Whitney, p = 0.0352). Comparing the infestation in the three palm trees species globally, we can observe a significant difference between them (Kruskal-Wallis, p = 0.015). The results show greater triatomine density in babaçu and uricuri palm trees (Mann–Whitney, p < 0.05) compared to the inajá palm tree*.*

**Table 3:** Number of triatomines collected by collection period, community and palm tree species

**Community/Palm tree**	**Araipá**		**Nova estrela**	**São tomé**		**Total**
	**2008**	**2009**	**2008**	**2008**	**2009**	
*Attalea speciosa*	0	0	45	88	93	226 (30.4%)
*Attalea maripa*	66	5	1	36	93	200 (26.9%)
*Attalea phalerata*	0	0	112	77	128	317 (42.7%)
Total	66	5	158	200	314	743 (100%)

In the second collection, we found 264 (82.8%) nymphs and 55 (17.2%) adults of two triatomine species, *R. robustus* and *R. pictipes* in the two communities surveyed: Araipá and São Tomé. The statistical analysis showed significant difference of triatomine density between the two communities (Mann-Whitney, p = 0.0005), again with greater density of triatomines in São Tomé palm trees. The triatomine density in 2009 was similar to that obtained in 2008. Statistically, we have found global differences between the three species of palm trees (Kruskal-Wallis, p = 0.000), with the inajá palm trees presenting a lower density of barber bugs (Mann–Whitney, p < 0.05).

### Molecular identification of the specimens collected

The results obtained through the Cytochrome B analysis of a sample of *R. robustus* collected, corroborate the identification made by the classification keys proposed [3], confirming the taxon found. All *R. robustus* presented 99% similarity with haplotype roBR3m, from Apuí, Amazonas, corresponding to the group *R. robustus* III [16].

The Triatominae collected were accounted by palm tree species and sampling year, and the Table 4 presents triatomine population density data in each community, in relation to the collection period, palm tree species and development stage of the insects.

**Table 4:** Density of triatomines collected on each community, in relation to the collection period, palm tree species and evolutionary stage of the insects

**Evolutionary stage**	**2008**							**2009**				**Total**
	**Araipá**	**Nova estrela**			**São tomé**			**Araipá**	**São tomé**			
	**Inajá**	**Babaçu**	**Inajá**	**Uricuri**	**Babaçu**	**Inajá**	**Uricuri**	**Inajá**	**Babaçu**	**Inajá**	**Uricuri**	
1st	0	8	0	10	8	2	4	0	11	11	20	74
2nd	10	17	0	15	7	2	9	0	19	11	25	115
3rd	17	9	1	16	20	6	22	0	16	24	31	162
4th	13	3	0	22	17	14	30	2	11	24	18	154
5th	13	3	0	21	22	8	11	1	15	12	13	118
Female	4	1	0	8	2	1	1	1	8	4	5	35
Male	9	4	0	20	12	3	0	1	13	7	16	85
Total	66	45	1	112	88	36	77	5	93	93	128	743

Inajá = *Attalea maripa*; Babaçu = *Attalea speciosa*; Uricuri = *Attalea phalerata.*

### Infection by Trypanosomatids

#### Direct parasitological examination and hemolymph examination

The direct stool parasitological examination for confirmation of infection by trypanosomatids was performed only on the triatomines that arrived alive at LATEC. Therefore, in the first collection, only 32 (7.5%) of the 424 triatomines collected were examined and none of the insects presented flagellate forms of trypanosomatids. In the second field collection, 63 (19.7%) of the 319 triatomines collected were examined, and 21 triatomines were infected, all from the São Tomé community. The global data indicates that 21 of the 96 triatomines examined were infected, generating a global index of 21.9%.

The insects examined (n = 96) were also submitted to hemolyph examination in order to confirm the infection by *T. rangeli*, however, we were not able to find the parasite in any of the triatomines examined. Table 5 presents the global collection data, triatomine examination, number of triatomines infected and percentage value of barber bugs infected.

**Table 5:** Number of triatomines collected, examined, infected with trypanosomatids, by communities, palm tree species and collection period

**Palm tree**	**Triatomines collected**					**Triatomines examined**					**Triatomines infected**				
	**Araipá**		**São tomé**		**Nova estrela**	**Araipá**		**São tomé**		**Nova estrela**	**Araipá**		**São tomé**		**Nova estrela**
	**2008**	**2009**	**2008**	**2009**	**2008**	**2008**	**2009**	**2008**	**2009**	**2008**	**2008**	**2009**	**2008**	**2009**	**2008**
*Attalea speciosa*	0	0	88	93	45	0	0	19	21	0	0	0	0	9	0
*Attalea maripa*	66	5	35	93	1	7	0	6	20	0	0	0	0	3	0
*Attalea phalerata*	0	0	77	128	112	0	0	0	22	1	0	0	0	9	0
Total	66	5	200	314	158	7	0	25	63	1	0	0	0	21	0

#### Identification of natural infection of *Rhodnius* spp by *Trypanosoma cruzi* through molecular analysis

We examined 740 of the 742 *Rhodnius* spp*.* collected. In the first field collection, 32 (7,6%) of the 423 *Rhodnius* spp*.* collected amplified a fragment of 350 bp corresponding to the same size of the Colombian strain, characteristic of *T. cruzi* type 1 [22]. These infected triatomines were distributed in three species of palm trees (babaçu, maripa and uricuri) and were present in all the developmental stages. Araipá was the only community that did not present triatomines infected by *T. cruzi* and the statistical results did not show a significant difference of the number of insects infected between Nova Estrela and São Tomé (Chi-square, p = 0,4506). Taking into account the species of palm trees surveyed in the three communities, only the inajá palm trees differed statistically from the uricuris (Fisher’s exact test, p = 0.0261).

In the second field collection, conducted in 2009, out of the 319 triatomines collected, we investigated the natural infection in 317 insects and found infection in 93 (29.3%) *R. robustus*. The three specimens of *R. pictipes* collected were not infected by *T. cruzi*. The global index of infection was 16.9% and, as in 2008, the only community that did not present triatomines infected by *T. cruzi* was Araipá. Considering both communities surveyed in 2009 (São Tomé and Nova Estrela), the babaçu palm trees presented a higher index of infection regarding the inajás and uricuris (Fisher exact text, p = 0.0000). In addition, the year of 2009 presented a higher rate of infection by *T. cruzi* when compared to the year of 2008 for each palm tree species (Fisher’s exact test, p < 0.03247).

#### Identification of natural infection of *Rhodnius spp* by *Trypanosoma rangeli* through molecular analysis

Sixteen triatomines of the 423 *Rhodnius* spp. collected in 2008 amplified a fragment of 620 bp confirming the presence of infection by *T. rangeli*. Infection was found in all stages, except the first stage nymphs. Six of the 76 palm trees dissected contained insects infected by *T. rangeli*; five *Attalea phalerata* palm trees in the community of Nova Estrela and one *A. maripa* palm tree in São Tomé. The triatomines collected in the Araipá community did not present infection, and neither did the babaçu palm trees. Statistical results showed significant difference between the communities, and the triatomines of Nova Estrela presented a higher rate of infection by *T. rangeli* (p < 0.00659). In addition, there was also a significant statistical difference of the infection of the triatomines by *T. rangeli* in the uricuri palm trees in 2008 (Fisher’s exact test, p < 0.0134).

In 2009, out of the 319 triatomines collected, 317 were evaluated and 52 *R. robustus* and one *R. pictipes* also amplified the fragment specific for *T. rangeli* (infection index of 16.7%). We found infection in all developmental stages. One of the uricuri palm trees from the community of São Tomé had 25 triatomines infected by *T. rangeli* of the 31 collected.

Ten of the 60 palm trees investigated in the year 2009, had triatomines infected by *T. rangeli*, six were uricuri palm trees, two were babaçu palm trees and two were inajá palm trees, all belonging to the São Tomé community. We did not find triatomines infected by *T. rangeli* in the Araipá community. Considering the two communities surveyed in 2009, we found a significant statistical difference of triatomine infection by *T. rangeli* (p = 0.00001). However, the results of the comparison made with the three palm tree species did not indicate any statistical difference (Fisher’s exact test, p ≥ 0.4975).

In total, considering the infection data of 2008 and 2009, we found statistical differences only between the babaçu and uricuri palm trees (Chi-square, p = 0.035). In addition, the data of infection by *T. rangeli* presented statistical differences between the two years surveyed only in the babaçu palm trees, demonstrating greater infection of *Rhodnius* in the year 2009 (Fisher’s exact Test, p = 0.01105).

### Mixed infection

When crossing the data of natural infection of *T. cruzi*, using the non-transcribed intergenic spacer of the mini exon gene, with the data of the natural infection by *T. rangeli* using the repetitive gene of small nucleolar RNA Cl1 (sno-RNA-Cl1), it was possible to verify which triatomines had a mixed infection, i.e. the presence of the two parasites in the same vector insect.

In 2008, we found five triatomines with mixed infections, three males and two fourth stage nymphs. These triatomines were distributed in five palm trees, four uricuri palm trees in the Nova Estrela community and one inajá palm tree in São Tomé.

In the year 2009, nine triatomines presented mixed infection, all from the community of São Tomé. Mixed infections were found in all developmental stages, except in first and second stage nymphs. Table 6 presents the mixed infection data distributed by palm tree species in the year 2009.

**Table 6:** **Number of triatomines with mixed infection (presence of *T. cruzi *** **and *T. rangeli*) detected via PCR, 2009**

**Palm tree**	**São tomé**							**Total**
	**Evolutionary stage**							
	**Male**	**Female**	**5th**	**4th**	**3rd**	**2nd**	**1st**	
Babaçu	0	2	1	0	0	0	0	3
Inajá	2	1	0	0	0	0	0	3
Uricuri	0	1	1	2	4	0	0	8
Total	2	4	2	2	4	0	0	14

## Discussion

The Amazon was, for a long time, considered a risk-free area for *T. cruzi* transmission. However, after the 60s, with the construction of the Trans-Amazonian Highway and of the BR-163 (Cuiabá-Santarém), the migration of people, mainly from the northeast, to the Brazilian Amazon Region has grown alarmingly. Among the consequences generated by the increase in the Amazonian population, we can mention the environmental changes and the emergence (or re-emergence) of diseases. It was only after 1969 that attentions were focused on the Amazon, with the report of the first acute case of American Trypanosomiasis from Belém, Pará [8]. Nevertheless, in the late 20th century and early 21st, there were reports of new forms of infection by *T. cruzi* in the Amazonian region, associated with the presence of triatomines in houses without colonization and oral transmission by contaminated food [10,23,24]. It should be emphasized that deforestation for deployment of extensive farming, monocropping, logging, agrarian colonization and migrations of populations associated with the process of urbanization in the region are important factors for the emergence of disease [23].

Much of the Amazonian territory comprises rural communities and areas, and subsistence agriculture is the main source of food for these people. In these areas, the practice of cultivation after burning is common. One of the consequences of this is the proliferation of palm trees, which are ecotopes to triatomines, mainly of the genus *Rhodnius*. The palm trees produce about 15 tons of dry organic matter, value far superior to the other species of trees [25]. In addition, palm tree species recover much more quickly after the fire than other plant species present in the area. All the communities surveyed use this burning practice. In regions already deforested, which serve as pasture for cattle, the only plant species of medium and/or large size found was the palm tree.

Although geographically the three communities are relatively close to each other, each one has a different history. São Tomé, found near the Tapajós River, is the oldest community, with at least 64 years of existence. It is part of the municipality of Aveiro and its population comprises almost exclusively people from the state of Pará, from the Tapajós Region. Women are engaged in domestic activities, while men work with fishing and family farming [26]. The riverside portion of São Tomé comprises primarily grasslands, however, the presence of forest fragments within the community is larger in relation to other communities. Due to its years of existence, São Tomé is the community that appears to be the most stable, with less anthropic pressure on the landscape.

Nova Estrela, close to the Trans-Amazonian Highway, is part of the municipality of Rurópolis. It is a typical settlement of immigrants, mostly northeasterners, from the State of Maranhão. This community does not have contact with the Tapajós River and the activities of the inhabitants are very similar to São Tomé, except for the fishing. It is believed that the community was established between the 70s and 80s, a period of implementation of the Trans-Amazonian Highway [26]. Due to construction of the Trans-Amazonian Highway and the BR-163, associated with the process of territorial expansion and occupation of land, the environment of Nova Estrela was probably highly transformed, and today there is a predominance of pastures, short grass and small fragments of secondary forests.

The third community, Agrovila-Araipá, near the Araipá Lake, comprises, mostly, people from the States of Pará and Maranhão. Men are engaged in fishing and cultivating mainly rice, beans and cassava for maintenance of their families. Araipá is the community with the greatest human pressure on the environment. The landscape in Araipá is strongly anthropized, with predominance of pastures.

The tops of the palm trees form a very particular ecosystem that serves as shelter for a huge range of vertebrate animals, which are sources of nourishment for the vectors of Chagas’ disease [6]. It is also worth mentioning that the various species of palm trees are an important economic resource for the residents of the Brazilian Amazon and of other regions of Brazil. The inhabitants remove the leaves from the trees and use them for handcrafts and as roofs for their houses. They also collect the fruits and extract the palm heart for nourishment, in addition to the stem and seeds. Thus, the extractive activities of palm trees should be considered a risk of infection by *T. cruzi* to humans, as already demonstrated in the vicinity of the Rio Negro, in the municipality of Barcelos, Amazonas [27].

The results of infestation showed, globally, that more than half of the palm trees were infested by triatomines. The community with the lowest rates of infestation was Araipá. Almost all the palm trees surveyed in Araipá were close to young short grass but not pastures and presented marks of fire on the stem and around the top. The use of fire by the inhabitants is common, which has possibly interfered in the values of infestation and even on the triatomine density. Although it was also possible to observe fire marks in the palm trees of Nova Estrela, the landscape of this community appears to be less anthropized, with greater presence of short grass and vertebrate animals associated with more diversified palm trees. Nova Estrela was the only community that was surveyed in the period coinciding with the end of the dry season in 2008 and not 2009, due to the lack of local infrastructure and difficulty of locomotion to conduct the study a second time.

On the other hand, São Tomé seems to be the most stable community with less anthropic impact. There, the presence of forest fragments, old short grass and pastures is constant. Although we observed fire marks in the palm trees, this practice is less common. In a pasture which presented a great number of infested palm trees, a high density of triatomines and a large diversity of vertebrates in the palm trees, the last burnt had been performed more than five years ago. Therefore, we noticed that, although the use of fire appears to interfere negatively in the population of triatomines, the partial re-establishment of the palm tree top ecosystem took less than five years.

The *Attalea* genus palm trees have been investigated for many years as ecotopes of triatomines [3,5,6,28]. The architecture of the de *A. speciosa* top favors the colonization of triatomines, allowing greater microclimatic stability and serving as a shelter for several species of animals [6], including large vertebrates, such as *T. tetradactyla* Linnaeus, 1758 [29]. With all this complexity, the palm trees laid out a five-level trophic network comprising the different species of animals that inhabit the palm trees [25]. In Panama, due to the large triatomine density found in *A. butyracea*, it was pointed out as a natural ecotope to *R. pallescens* Barber, 1932 [5]. In addition, this palm trees species was considered as an ecological indicator of risk areas for Chagas disease. *A butyracea* had an average of 21 *R. pallescens* against 1.6 in *Copernicia tectorum* (Kunth) Mart., species of palm tree with the second highest density of triatomines. In the Tapajós region, three species of *Attalea* were investigated. Statistical results showed lower density of triatomines in *A. maripa*, mainly due to the low number of palm trees infested and to the low triatomine density present in Araipá. None of the *A. aculeatum* (tucumã) investigated were infested by triatomines. Beside it has been reported as an ecotope for triatomines in the Amazon Basin [25], it is less frequent in the Tapajós region and the sample investigated was very limited.

Still with respect to triatomine density, there was a predominance of nymphs in the palm trees. Only 120 (16.1%) of the 743 triatomines collected were adults. A curious fact was the amount of males found (n = 85) compared to females (n = 35) in the palm trees. In fact, data from 1985 and 1986 from the epidemiological surveillance of the State of São Paulo show that there is greater intradomiciliary infestation by fertilized females capable of forming colonies [30]. On the other hand, the study of flight behavior has shown that *T. sordida* is capable of flying more than 100 meters [31]. In another study, females of *T. infestans* begin flying more often than males of the same species [32]. In this same study, the authors found that *R. prolixus* has a greater predisposition to fly when compared with *T. infestans*[32]; this behavior can be related to different habitats where we can find this species. The flight and the nutritional status of two species of *Triatoma* were assessed and it was found that the females collected in light traps presented more prolonged fasting compared to females found in natural ecotopes [33]. In addition, as discussed [32], if the flight is a dispersion/colonization mechanism of the triatomines, females are more likely to fly than males. It is worth mentioning that the only triatomine specimen found inside a house in Nova Estrela was a *R. robustus* female that was in a long fast. Thus, a factor that may explain the low number of females in the palm trees of the middle Tapajós must be the active dispersion for forming colonies or even for food searching attracted by lights at night.

In this study, due to great mortality of the triatomines collected, the typing and identification of *T. cruzi* has been performed without the isolation of the strain, i.e., direct from the dead triatomine. Thus, there was no type of selection of the parasite populations. In addition, the positive and negative controls used in the PCR reactions for the identification of natural infection by *T. cruzi* and *T. rangeli* were critical. The markers used were specific to each of the parasites, providing, thus, reliable results of high rates of infection. *Tamandua tetradactyla* is considered a host of the *T. rangeli* in the region of study [29]. Due to the size of this animal that inhabiting the tops of the palm trees, the sylvatic cycle of this parasite will be constant. In addition, the fact that other animals inhabit the palm trees concurrently with the triatomines, reinforces the maintenance of the sylvatic cycle of trypanosomatids. The presence of *Didelphis marsupialis* in the region of study draws our attention since it is a vertebrate considered to have high rates of *T. cruzi* infection [34], also, it presents metacyclogenesis of the *T. cruzi* in its anal glands. In the region studied, the results of infection demonstrated the propagation of *T. cruzi* with *T. rangeli* in the same vector insect. The constant presence of *T. rangeli* in the region is a fact that deserves attention because this parasite shares surface antigens with *T. cruzi*. In serological surveys, the presence of *T. rangeli* may cause false positive results [35,36].

The results presented here demonstrated the existence of a large population of *R. robustus* in the middle Tapajós Region, Pará, Brazil. It was possible to observe that the different forms of use of the soil interfere with the infestation of palm trees and triatomimes density: negatively, with the use of fire, and, positively, with the preservation of the environment. In addition, data showed the maintenance of the sylvatic cycle of *T. cruzi*, even with the presence of mixed infections in the same vector. These findings indicate a potential connection between sylvatic and domestic cycles of transmission of Chagas disease, as already demonstrated in the Ecuadorian Amazon [37,38], which has not yet been consolidated in this region of the Tapajós probably due to the little capacity for colonization of the artificial environment by local vectors. In the period analyzed, we only found one adult specimen inside a house. The State of Pará has been experiencing an increasing number of acute outbreaks of human infection by *T. cruzi* associated with oral transmission, usually linked to the consumption of contaminated bacaba and acai berry juice [10,23,36]. The açaí palm is rarely present in the list of palms of the region here studied. The same is true for the bacaba, since it is very rarely seen in the area, and it is also not used in food. Not far from there, however, in the municipality of Santarém, in 2006, 17 acute cases with transmission associated with the consumption of the juice of bacaba were reported [36].

## Conclusions

The existence of an intense sylvatic cycle demands surveillance to prevent the transmission, either by the occasional presence of triatomines inside the houses, or by food contamination. Furthermore, the difficulties of locomotion, the absence of an effective monitoring control program in the region associated with the lack of a health service structure and basic medical assistance around the communities, are primary factors that must be assessed with urgency in order to maintain the health and well-being of these Amazonian populations. Moreover, the information of the communities about American Trypanosomiasis may help to prevent the risk of transmission.

### Financial support

This work was carried out within the framework of the PLUPH (Poor Land Use, Poor Health) international project, with support from the CPqRR/FIOCRUZ (Brazil) and the Global Health Research Initiative (GHRI), a collaborative research funding partnership of the Canadian Institutes of Health Research, the Canadian International Development Agency, Health Canada, the International Development Research Centre, and the Public Health Agency of Canada.

### Ethical statement

This study strictly followed the ethical codes of Brazil. The sampling was carried out in full compliance with the Brazilian laws (IBAMA, license number 16485–2).

## Competing interest

No conflict of interest related to this article for all authors.

## Authors’ contributions

Financial Support: CAR, RD, FM, ML. PLUPH Project Global Design: CAR, RD, FM, ML, LD, MH. Conceived the study: FBSD, MQ, CAR, LD, MH. Collected the bugs: FBSD, MQ, GM. Prepared samples: FBSD, MQ. Analyzed data: FBSD, MH, LD, CAR. Interpreted data: FBSD, CAR, MH, LD. Statistical Analysis: ACLL. Wrote the manuscript: FBSD, CAR, LD. All authors read and approved the final version of the manuscript.
